# Outcome and complications after surgery for thyroid carcinoma in pediatric age—an evaluation of practice

**DOI:** 10.1186/s12957-022-02757-1

**Published:** 2022-09-14

**Authors:** Ahmed Elgendy, Emad M. Shehata, Sherif M. Shehata

**Affiliations:** 1 Surgical Oncology Unit, Faculty of Medicine, Tanta University, 35 Ali Beek Elkbeer street, Tanta, 31515 Egypt; 2 Otolaryngology Department, Faculty of Medicine, Tanta University, Tanta, Egypt; 3 Pediatric Surgery Department, Faculty of Medicine, Tanta University, Tanta, Egypt

**Keywords:** Thyroid carcinoma, Pediatric, Thyroidectomy, Complications, Outcome

## Abstract

**Objectives:**

To discuss management protocol, surgical complications, and outcomes of thyroid carcinoma in children.

**Methods:**

We performed a retrospective analysis including all pediatric patients with thyroid carcinoma who were managed at our institution between January 2011 and January 2021. Data were analyzed regarding demographics, clinical features, operative details, postoperative complications, and survival data.

**Results:**

Thirty-two patients were identified; 26 females (81.25%) and 6 males (18.75%). The median age at operation was 14 years (range: 5–18). Twenty-six (81.25%) patients presented with palpable thyroid swelling. Median tumor size was 3 cm (range: 1–7). Metastatic workup did not detect any pulmonary metastases. Total thyroidectomy was performed in 25 patients (78%), and 16 of them underwent additional bilateral neck dissection (16 had central nodal dissection, and 7 had both central and lateral nodal dissection). Seven patients (22%) underwent hemithyroidectomy, and only one of them had a completion thyroidectomy after 2 weeks. Conservative resection was adopted in six children with similar criteria (tumor size < 1.5 cm in one lobe, no extrathyroid extension, differentiated thyroid carcinoma, no detected lymph nodes). Postoperative complications occurred in eight patients (all had total thyroidectomy) with an overall incidence of 25%. Seven patients had transient morbidities that were managed conservatively (chylous leak *n* = 1, hypoparathyroidism *n* = 3, and nerve palsy *n* = 3). At a median follow-up time of 54 months, four patients had relapsed (all underwent total thyroidectomy). The 5-year OS and EFS were 100% and 87.5%, respectively.

**Conclusion:**

Operative resection for pediatric thyroid carcinoma can be performed with average short-term complications and achieving excellent outcomes. Total thyroidectomy remains the standard procedure of choice in the majority of those patients. However, conservative surgery can be successfully adopted in a well-selected group of children with favorable long-term results as per our findings.

## Introduction

Thyroid carcinomas among the pediatric population are rare tumors that represent only 0.7% of all encountered childhood malignancies [1, 2]. Their incidence was approximately estimated to be 0.4–0.7 patients per 100,000 children [3]. The frequency of such neoplasms increases with age, and therefore, they are the eighth most common malignant entities in adolescent age [4].

Children who are afflicted with these tumors usually present with palpable thyroid nodules, with or without cervical lymphadenopathy. However, some patients can be referred due to enlarged cervical nodes only. Other patients can be detected incidentally during evaluation of another disease or even discovered surprisingly as a pathological finding after resection of a presumed benign lesion [4, 5].

Patients within the pediatric age group often had different clinical characteristics including more advanced disease and a higher rate of recurrence, when compared with their adult counterparts [6]. Despite the aggressive presentation of thyroid carcinomas in children, better survival outcome was achieved when compared to adult patients due to favorable tumor biology [7]. Consequently, these cancers in children and adolescents should be managed by a special approach to avoid either insufficient or excessive treatment [8].

Surgery with complete tumor excision is the cornerstone for a realistic chance of long-term cure. However, the optimal surgical strategy regarding the extent of resection and lymph node management is still a debatable issue in the literature [9]. This may be due to the paucity of these entities in childhood and potentially serious surgical complications that could impact the operative decision-making especially with non-specialized surgeons [4, 7, 9, 10]. The current study discusses management protocol, surgical complications, and outcomes of thyroid carcinoma in the pediatric population.

## Material and methods

This study contained all children and adolescents with thyroid carcinoma who were treated at our institution and affiliated regional centers. Patients older than 18 years of age or who diagnosed with benign thyroid tumors were excluded from the analysis. The surgical team included a consultant oncology surgeon (AE), a consultant in otolaryngology and head and neck surgery (EMS), and a consultant pediatric surgeon (SMS). The authors retrospectively reviewed their database during 10 years between January 2011 and January 2021. The data were analyzed regarding demographics of patients, clinical features, diagnostic aspects, operative details, postoperative complications, final pathological results, and last follow-up and survival data. Our institutional review board approved the study design (IRB0010038—approval code: 34583/3/21). All patients’ parents signed a consent letter for surgery and data use in scientific publication at the time of management.

All included patients were initially evaluated by a neck ultrasound and thyroid function tests. Fine needle aspiration cytology from palpable nodules was conducted thereafter to reveal a primary pathological diagnosis according to the Bethesda system which was described in 2007 [11]. Computed tomography (CT) on the neck was conducted in selected children based on the need for further details after the initial assessment. Chest CT was done for all children to exclude the presence of lung metastases. Surgery, using a conservative resection (hemithyroidectomy) or a radical approach (total thyroidectomy), was performed for all patients. Lymph node management was tailored according to clinical, radiological, and operative findings. Surgical strategies and administration of adjuvant radioactive iodine (RAI) were adopted as per formerly published guidelines [8, 9, 12, 13].

Complications after surgical resection were categorized according to Clavien-Dindo classification [14]. Transient or permanent hypoparathyroidism, hypocalcemia, and recurrent laryngeal nerve injury were assigned as post-procedural morbidities. Circulating levels of parathormone and calcium were assessed few days after surgery. The nerve condition was based on examination of vocal cords during extubation by the attending anesthesiologist and the patient's voice after recovery. Clinical evaluation, thyroid hormones assay, neck imaging, and body scanning were performed in the follow-up period to detect any recurrences. Follow-up visits were scheduled 2 months after surgery, then every 6 months in the first 2 years and every year thereafter. Data regarding relapses and deaths were registered till January 2022.

Patients’ notes were extracted and collated in an excel sheet. Patients’ characteristics and clinical factors were summarized by descriptive statistics using Statistical Package for Social Science (SPSS). Kaplan-Meier method was used to obtain the overall (OS) and event-free survival (EFS) curves. An event was assigned with the occurrence of tumor recurrence or death whichever happened first.

## Results

We identified 32 patients that were managed during the study period. Out of them, there were 26 females (81.25%). The median age at the time of operation was 14 years (range: 5–18 years). Twenty-four patients (75%) were in post-pubertal age group (older than 11 years). Seven children (21.9%) had a positive family history of thyroid diseases that required treatment, and two of them were sisters with familial medullary thyroid carcinoma (FMTC). None of the included patients had a relevant history of previous neck radiotherapy. Regarding the mode of presentation; twenty-six patients (81.25%) presented with palpable thyroid swelling, whereas cervical lymphadenopathy was the presenting symptom in four children (12.5%). Incidental diagnosis was encountered in two patients (6.25%) during neck ultrasound within several investigations due to other symptoms. The median tumor size was 3 cm (range: 1–7 cm) based on preoperative radiological measurement. Twenty-two patients (68.75%) had unilateral lesions, while multifocal tumors (more than one nodule in the same thyroid lobe by imaging) were detected in 17 cases (53%). None of the included patients had distant pulmonary metastases at the time of diagnosis. Table 1 shows demographics, preoperative imaging, and tumors’ criteria.

**Table 1:** The demographics, preoperative imaging, and tumors’ criteria

Parameter	Number (%)
**Gender**	
Female	26 (81.25%)
Male	6 (18.75%)
**Age**	
Pre-pubertal	8 (25%)
Post-pubertal	24 (75%)
**Imaging**	
US	32 (100%)
CT	27 (84.4%)
MRI	5 (15.6%)
**Tumor side**	
Unilateral	22 (68.75%)
*Left*	13
*Right*	9
Bilateral	10 (31.75%)
**Tumor focality**	
Unifocal	15 (47%)
Multifocal	17 (53%)
**Detected enlarged cervical lymph nodes**	
Yes	16 (50%)
*Clinically palpable*	4
*Radiologically detected*	12
No	16 (50%)
**Initial preoperative pathology (according to FNAC)**	
Non-diagnostic	-
Benign	1 (3%)
Atypical	3 (9%)
Suspicious follicular neoplasm	6 (18.75%)
Suspicious	12 (37.5%)
Malignant	10 (31.75%)

*US* ultrasound, *CT* computed tomography, *MRI* magnetic resonance imaging, *FNAC* fine needle aspiration cytology

**Fig. 1:**
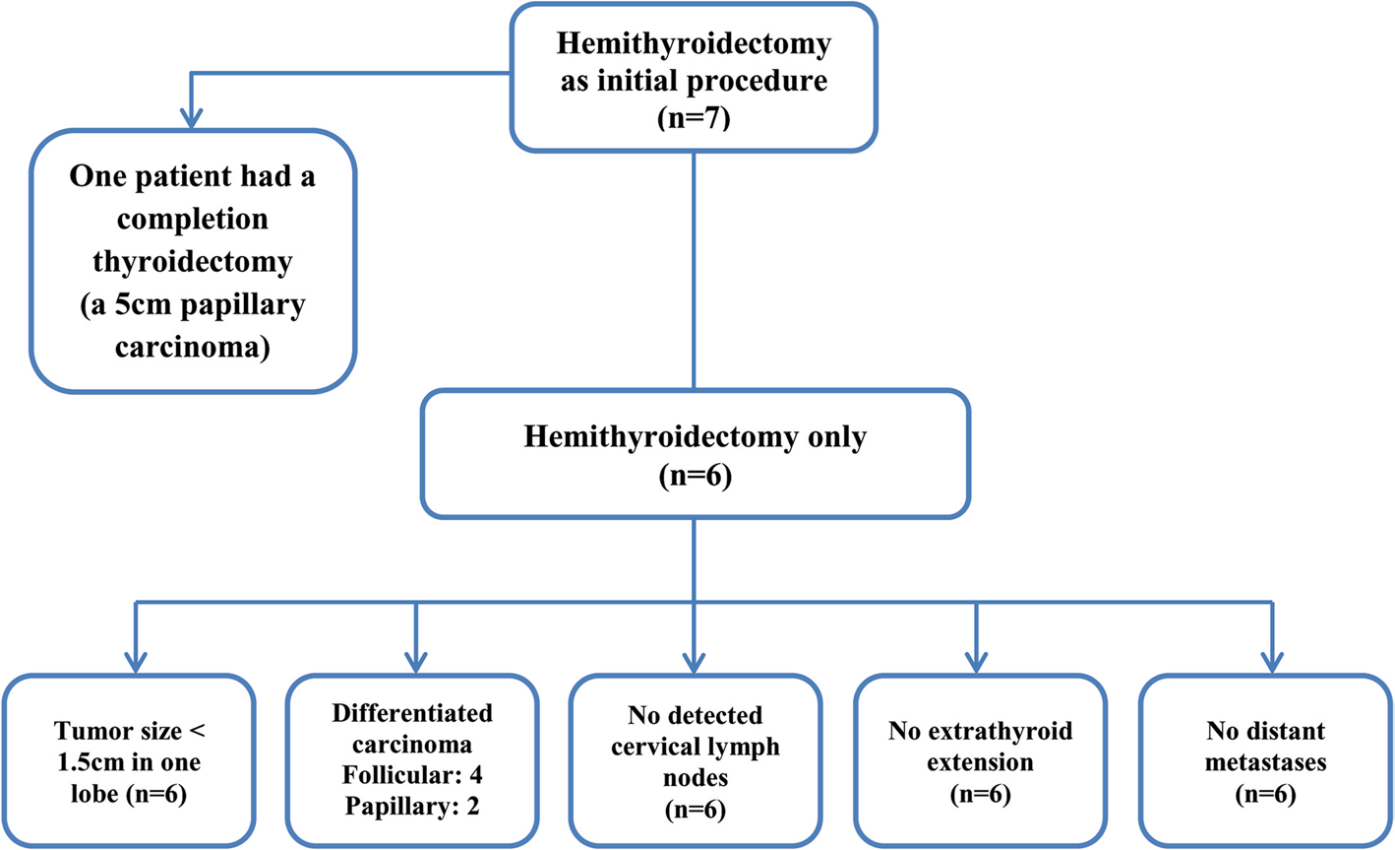
The criteria for the selection of the patients for conservative surgery

Concerning the extent of resection, total thyroidectomy was performed as a first procedure in 25 patients (78%), and 16 of them had additional bilateral neck dissection (16 had central nodal dissection, and 7 had both central and lateral nodal dissection). All children who underwent neck dissection had positive lymph nodes. The remaining seven patients (22%) underwent hemithyroidectomy without further neck dissection; one of them had such conservative resection due to an initial preoperative diagnosis of benign lesion (a 5-cm nodule). Completion thyroidectomy without neck dissection was conducted in this patient after 2 weeks as the final pathological result revealed follicular variant of papillary carcinoma. Papillary carcinoma represented the majority of pathological types (75%, 24/32) in all included patients. The six children who were only managed by conservative approach had differentiated carcinoma in postoperative pathological reports (follicular: 4–papillary: 2), and they had similar other tumors’ characteristics. The criteria for selecting conservative surgery among the patients were demonstrated in Fig. 1. Thirty patients (93.75%) had a complete tumor resection with a negative margin (R0). Vascular infiltration was not encountered in any patient. All patients had drain insertion after the surgical procedure. Table 2 summarizes the details of surgical management and final pathological results in all cases.

**Table 2:** The details of surgical management and final pathological results in all cases

Parameter	Number (%)
**Initial operative management**	
Total thyroidectomy (TT)	25 (78%)
*TT only*	9
*TT and node dissection*	16
*TT and CND*	16
*TT and CLND*	7
Hemithyroidectomy	7 (22%)
**Completion thyroidectomy**	
Yes	1/7 (14.3%)
No	6/7 (85.7%)
**Resection margin**	
R0	30 (93.75%)
R1	2 (6.25%)
R2	-
**Duration of drain**	
One day	15 (47%)
1–2 days	10 (31.25%)
2–3 days	6 (18.75%)
One week	1 (3%)
**Parathyroid glands**	
4 left in place	16 (50%)
2 left in place	10 (31.25%)
1 left in place	6 (18.75%)
**Final pathological results**	
Papillary carcinoma	24 (75%)
Follicular carcinoma	6 (18.75%)
Medullary carcinoma	2 (6.25%)
**Tumor size**	
> 1.5 cm.	26 (81.25%)
< 1.5 cm.	6 (18.75%)
**Extrathyroid extension**	
No	27 (84.4%)
Yes	5 (15.6%)
**RLN invasion**	
No	31 (97%)
Yes	1 (3%)

*CND* central node dissection, *CLND* central and lateral node dissection, *R0* negative resection margin, *R1* microscopic positive margin, *R2* macroscopic positive margin, *RLN* recurrent laryngeal nerve

Postoperative complications occurred in eight patients (all had total thyroidectomy) with an overall incidence of 25%. One case developed chylous leak after neck dissection that was managed conservatively and with stopping of enteral feeding and completely resolved after 1 week (grade I Clavien-Dindo). Transient hypoparathyroidism happened in three patients and was successfully managed within 3 weeks by administration of calcium supplementations and activated analogs of vitamin D (grade II Clavien-Dindo). No permanent hypoparathyroidism was reported in any case. Four patients were afflicted with recurrent laryngeal nerve morbidities; three of them had unilateral transient impairment of mobility that improved after 4 weeks on a course of corticosteroids (grade II Clavien-Dindo). The remaining child had bilateral nerve injury and paralysis as the tumor was invading the nerves bilaterally and required an urgent tracheostomy procedure. This patient underwent posterior cordopexy using laser technology 4 months thereafter by the second author, EMS for weaning from the tracheostomy tube (grade IIIb Clavien-Dindo). There were no complications encountered regarding postoperative bleeding, hematoma, or wound infection. All patients who had radical resection (except the two with medullary carcinoma) were referred to receive RAI as adjuvant therapy.

**Fig. 2:**
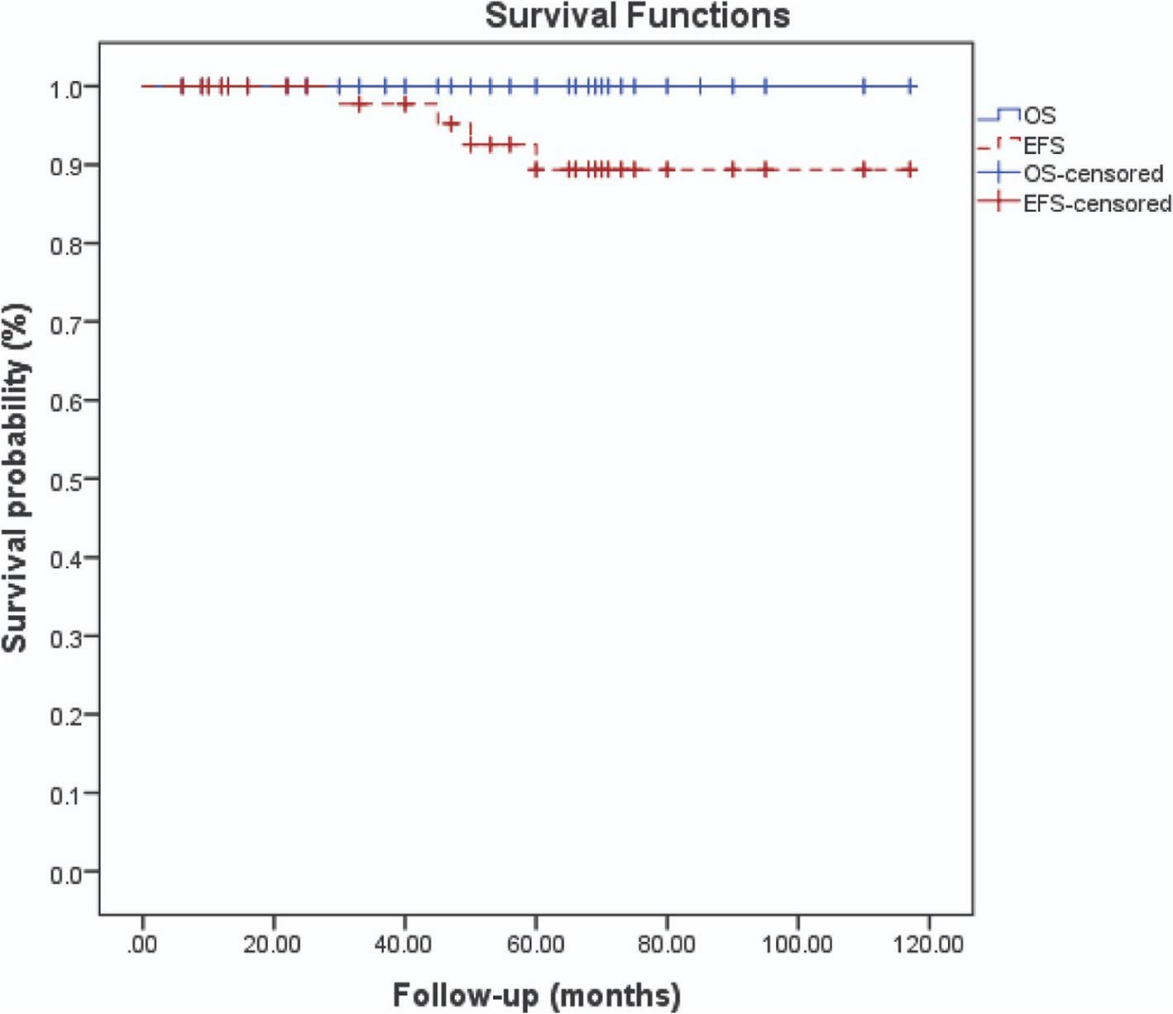
The 5-year OS and EFS in the included patients

The median follow-up time was 54 months (range: 9–117 months) with 95% CI (39.7–72.3). Tumor relapse occurred in four patients (all underwent total thyroidectomy) with a rate of 12.5% [95% CI (9.5–16)]. One of them had an isthmus nodule and the other patient had a nodal recurrence. Both patients underwent secondary surgery for resection of the recurrent lesion or neck dissection followed by further doses of RAI therapy. The remaining two cases developed relapses in the thyroid bed; however, they were entirely treated by RAI. There were neither distant relapses nor second malignancies detected among all patients. However, one girl had an ovarian benign cystic teratoma 3 years after total thyroidectomy that was managed using minimally invasive surgery by the senior author, SMS. All children were alive at the end of the follow-up period. The 5-year OS and EFS were 100% and 87.5%, respectively. The survival outcome in the included patients was reported in Fig. 2.

## Discussion

Thyroid lesions in the pediatric population are less prevalent when compared to adults; however, such nodules in children are more likely to be malignant [15]. Undoubtedly, surgical resection is the principal modality for the management of pediatric thyroid carcinoma; however, the extent of resection remains controversial in differentiated carcinomas. Total thyroidectomy versus hemithyroidectomy as a surgical choice is a matter of debate in children [16]. Several studies have declared that radical resection is the standard approach of choice to reduce the relapse rate and achieves favorable EFS [17–20]. Conversely, some investigators support conservative surgery in unilateral tumors arguing that the radical approach does not significantly improve the outcome in addition to avoid serious postoperative complications that may occur during total thyroidectomy [1, 21, 22].

The current study highlights the surgical protocol for these tumors and reveals that the extent of resection can be customized as per the patient’s criteria. We adopted total thyroidectomy in the majority of our patients, while hemithyroidectomy was safely performed in diligently selected children with rigid follow-up. The conservative surgery was exclusively adopted in the patients with the following criteria: unilateral lesions of differentiated thyroid carcinoma (size less than 1.5 cm) with no extrathyroid extension, no detected lymph nodes, and no distant metastases. There was no recurrence encountered among the patients who underwent a conservative resection. Similarly, two studies reported that hemithyroidectomy in carefully selected patients achieved a relapse rate of 1% and 3.4%, respectively by Pacini and Spinelli et al. [6, 23]. Interestingly, the local recurrence rate in this study occurred in patients who had a radical resection and it was the same rate (12.5%) in a former study that adopted total thyroidectomy strategy for all patients [19]. On the other hand, Astle et al. demonstrated 100% recurrence-free survival using the radical resection in all children with thyroid carcinoma [17]. The authors of this study believe that the local relapse rate is not related to the operative extent of resection and it is mainly correlated with tumors’ characteristics and biology. Consequently, those patients should be treated on the basis of a tailored approach as a group of children can avoid lifetime hormonal replacement without any compromise in oncological outcomes. However, prospective cohorts are still required to compare different management strategies to recurrence rates and formulate absolute recommendations with a balance between benefits and risks.

In this study, an excellent OS was observed among all included children. Similarly, Babala et al., Spinelli et al., and de Jong et al. reported a 100% survival rate after complete surgical resection for all their patients [4, 6, 24]. Additionally, the American Pediatric Surgical Association (APSA) Cancer Committee declared in 2020 that 5-year OS in non-metastatic patients is usually around 99.8%, whereas in cases with distant metastases, the OS is about 97% [25]. Our results as well as the aforementioned published data support that favorable oncological outcomes regarding these tumors can be achieved after optimal surgical management and compliance with evidence-based guidelines.

Complications after surgical resection for pediatric thyroid carcinoma are not uncommon and are usually based on several factors including surgeons’ experience, the aggressiveness of maneuvers, and tumors’ characteristics. It was observed that surgical complications are more prevalent in children when compared to adult patients [26]. This could be attributed to the dissection of delicate structures in the pediatric age in addition to the increased sensibility of parathyroid glands regarding ischemic injury [27]. The overall complications rate in our study (25%) was in concordance with other published data among pediatric patients that reported rates ranged between 22 and 39% [4, 18, 24]. Slight differences between cohort studies can be explained due to variable sample sizes. Recurrent laryngeal nerve injury is considered major procedure-related morbidity that results in voice dysfunction or even aphonia. The analysis of published data declared that its percentage is from 10 to 40% in children [1, 28]. The rate of temporary neurapraxia in this study was 9%, and although this was higher than other reports that documented such events ranged between 2.8 and 4.8% [6, 29], all our patients were completely recovered. Permanent palsy occurred in 3% among our patients and similar favorable results (2.5% and 4.5%) were also reported by Wada et al. and Babala et al., respectively [4, 27].

Hypocalcemia is a commonly encountered complication after radical resection in pediatric age. It occurs either due to injury and/or devascularization of parathyroid glands during dissection and leads to postoperative muscle spasm or serious neurological features in severe, untreated cases [30]. The rate of post-thyroidectomy hypocalcemia in children was estimated to be about 12–37.5% [24, 28, 31]. In this study, transient hypocalcemia was encountered in 9% of patients. Other investigators, however, reported this temporary complication in 21%, 27.3%, and 41% of their patients [4, 32, 33]. Additionally, a recent systematic review in 2021 declared that the mean percentage from 15 studies was about 35.5% [30]. Permanent hypocalcemia did not happen in any patient within our study as well as another report [4], and it was only encountered in 1% and 3% of patients in previous studies [32, 33]. Eventually, the majority of surgical complications in our study as well as the aforementioned studies [4, 18, 24, 32, 33] were short-term, transient morbidities that were cured after conservative treatment and without any further sequelae. Consequently, we believe that surgical resection for such tumors can be safe especially when performed by a specialized, dedicated team. We know that one of the limitations in such type of studies to elaborate a general consensus from our results is the small sample size. However, this study has several advantages as there was no selection bias among cases, the operating team was the same during the study period, and there was no surgical variability over time.

## Conclusions

Operative resection for pediatric thyroid carcinoma can be performed with average, short-term complications and achieving excellent surgical and oncological outcomes. Total thyroidectomy remains the standard procedure of choice in the majority of those patients. However, conservative surgery can be successfully adopted in a well-selected group of children as per our findings to avoid lifetime hormonal replacement.

## Data Availability

The datasets used and/or analyzed during the current study are available from the corresponding author on a reasonable request.
